#  Lean: breaking down barriers for the sake of improvement

**DOI:** 10.1093/intqhc/mzz112

**Published:** 2019-12-22

**Authors:** Mark Graban

**Affiliations:** Taylor and Francis Group, Boca Raton, London, New York

Research estimates that healthcare organizations experience implementation failure in almost two-thirds of initiatives manifesting in the ‘quality chasm’ [1]. Although Lean Six Sigma methodologies have had mixed reviews in healthcare, ranging from the critical [2–4] to the highly supportive [5,6], I believe that this supplement demonstrates its appropriate use in healthcare settings, through appropriately prepared and supported inter and multidisciplinary teams, can help close this ‘quality chasm’.

Healthcare improvement quite often results from breaking down silos and crossing breaking barriers in many ways. Methodologies, practices and mindsets can be adopted (and adapted) from manufacturing to healthcare, as has occurred with Lean and Six Sigma over the past 20 years. People with varying backgrounds and perspectives can collaborate to create better, more affordable care—in Ireland and other countries around the world.

Healthcare professionals and leaders are incredibly motivated to improve—but there are many demands and priorities that compete for our attention. I was taught, by former Toyota leaders, to focus on safety, quality, on-time delivery, cost and morale. In healthcare, those priorities line up with (or strengthen) the need of people to improve safety (for patients and staff), quality (in terms of health outcomes and the patient experience) and access to care (shorter waiting times). Focusing on safety, quality and flow—combined with leaders and improvement facilitators who engage in the improvement process—leads to lower cost and better morale. The articles in this issue illustrate how focusing on quality and outcomes is perfectly compatible with healthcare organizations, driving results we can all be proud of. As McNamara and Teeling [7] note in their introductory paper on developing a university curriculum to support healthcare staff to implement Lean, Lean leaders recognize that many of the challenges they face are strategic and adaptive in nature, requiring the creation of conditions in which power is distributed and colleagues are engaged, empowered, enabled and educated to identify, challenge and address a range of structural, systemic and relational barriers and constraints.

Lean and Six Sigma will help us deliver lower cost and better value, especially when compared to traditional cost-cutting approaches. I was taught that lower cost is not the primary goal or objective—it is the end result of doing everything else well. Focusing too much on cost reduction can, unfortunately, lead people to make changes that might appear to reduce costs in the short-term, but with the unfortunate side effect of harming one of our other key measures. Or, short-term cost cutting in one department might increase costs in the long-term—or cost-cutting in one area might lead to higher costs for the system as a whole.

Lean teaches us to break down silos in our hospital or system. Lean emphasizes the end-to-end ‘value stream’ view. It can be very helpful to look beyond our local department because waste, errors and delays are often caused or exacerbated by poor handoffs between health professions or departments. There is no substitute for teamwork and better understanding the systems in which we work. We all benefit when cross-functional teams are formed and are given time to study how the work is done. Better understanding (and honestly admitting) our problems and opportunities is the first step in collaborating on improvements.

Worldwide, many healthcare organizations have started with a focus on improvement projects, as we read about in this issue. Improvement projects—and seeing people engaged in them—that is fantastic. But, Lean and Six Sigma projects have the limitation of using a handful of methodologies from approaches that are actually holistic systems. Projects are a wonderful first step, when healthcare professionals are engaged and they see that change is possible and that improvements can benefit all stakeholders.

Sometimes, this enthusiasm and spirit of improvement sadly ends with the conclusion of the project. Ideally, the initial sparks become an ongoing culture of continuous improvement. What does this require? Leadership. It requires that leaders embrace Lean and Six Sigma principles in their daily work. It means leading by example. It means continuing to ask—every single day—if there are opportunities or ideas for improvement. Leaders need to break down barriers to improvement. One of the reasons why the projects reported here were successful was the support of the Mater Lean Academy and the UCD School of Nursing, Midwifery & Health Systems over 6 years in the formation of Lean Leaders in Ireland and in developing and supporting a culture of improvement across the Irish healthcare system.

Lean, in particular, is widely admired and emulated because of its power as a management system and a culture across industries. Hopefully, projects are a first step in that direction, and I encourage all readers (and all contributing authors who follow) to continue on that path—establishing, nurturing, growing and sustaining a culture of patient- and staff-focused improvement.

In this supplement, you will read examples of organizations using a combination of Lean and Six Sigma methods. In particular, you will see references to a framework called DMAIC (Define, Measure, Analyze, Improve and Control)—a framework that comes from Six Sigma. Lean (and proceeding methods like Total Quality Management) uses a similar framework called PDCA (Plan, Do, Check, Act) or PDSA (Plan, Do, Study, Adjust). The terminology does not matter as much as the mindset of hypotheses and experiments. Every time we propose an improvement, our first steps should be considered a test. Perhaps the acronyms should be DMATC and PTSA, replacing ‘Improve’ and ‘Do’ with ‘Test.’ Maybe the ‘C’ in DMAIC should stand for ‘Continue Improving’ instead of ‘Control’ (the latter being a word that implies stability instead of further growth).

I started this article by talking about breaking down organizational boundaries. In our healthcare improvement efforts, we can also break down borders by sharing and learning from each other across different countries, cultures and continents. I have been fortunate to visit and work with hospitals across North America, Europe and Asia. I have met people who are doing similar work in Australia, Africa and South America. Our countries have different high-level systems for providing and paying for care. That said, the way patient care is delivered tends to be more similar than different. The way we manage and lead in healthcare also tends to be very similar. The good news is that lessons and inspiration can spread more broadly than our own country—and I expect the articles contributed to this issue will inspire others across Ireland, Europe and the planet.
